# Production of reactive species in alginate hydrogels for cold atmospheric plasma-based therapies

**DOI:** 10.1038/s41598-019-52673-w

**Published:** 2019-11-06

**Authors:** Cédric Labay, Inès Hamouda, Francesco Tampieri, Maria-Pau Ginebra, Cristina Canal

**Affiliations:** 1 Biomaterials, Biomechanics and Tissue Engineering Group, Dpt. Materials Science and Metallurgy, Universitat Politècnica de Catalunya (UPC), Escola d’Enginyeria Barcelona Est (EEBE), c/Eduard Maristany 14, 08019 Barcelona, Spain; 2 Barcelona Research Center in Multiscale Science and Engineering, UPC, Barcelona, Spain; 3 Research Centre for Biomedical Engineering (CREB), UPC, Barcelona, Spain; 4 Institute for Bioengineering of Catalonia (IBEC), Barcelona Institute of Science and Technology (BIST), c/Baldiri i Reixach 10-12, 08028 Barcelona, Spain

**Keywords:** Biomedical materials, Plasma physics

## Abstract

In the last years, great advances have been made in therapies based in cold atmospheric plasmas (CAP). CAP generate reactive oxygen and nitrogen species (RONS) which can be transferred to liquids. These CAP activated liquids display the same biological efficacy (i.e. on killing cancer cells) as CAP themselves, opening the door for minimally invasive therapies. However, injection of a liquid in the body results in fast diffusion due to extracellular fluids and blood flow. Therefore, the development of efficient vehicles which allow local confinement and delivery of RONS to the diseased site is a fundamental requirement. In this work, we investigate the generation of RONS (H_2_O_2_, NO_2_^−^, short-lived RONS) in alginate hydrogels by comparing two atmospheric pressure plasma jets: kINPen and a helium needle, at a range of plasma treatment conditions (time, gas flow, distance to the sample). The physic-chemical properties of the hydrogels remain unchanged by the plasma treatment, while the hydrogel shows several-fold larger capacity for generation of RONS than a typical isotonic saline solution. Part of the RONS are quickly released to a receptor media, so special attention has to be put on the design of hydrogels with *in-situ* crosslinking. Remarkably, the hydrogels show capacity for sustained release of the RONS. The plasma-treated hydrogels remain fully biocompatible (due the fact that the species generated by plasma are previously washed away), indicating that no cytotoxic modifications have occurred on the polymer. Moreover, the RONS generated in alginate solutions showed cytotoxic potential towards bone cancer cells. These results open the door for the use of hydrogel-based biomaterials in CAP-associated therapies.

## Introduction

Plasma is defined as a totally or partially ionized gas that contains a high number of reactive species, ions, electrons, metastable particles, etc. The development of plasma sources of small dimensions and able to operate at atmospheric pressure and at temperatures close to room temperature has fostered the development of a new field named Plasma medicine^1^. Atmospheric pressure plasma (APP) has been evaluated as an effective tool for sterilization^2^, cancer treatment^3^ or for enhancing wound healing^4^. APPs formed in air generate reactive oxygen and nitrogen species (RONS), which can be transferred to liquids through secondary reactions. Plasma-activated liquids (PAL) display different biological actions which have been mainly attributed to the generation of RONS such as hydrogen peroxides (H_2_O_2_), nitrites (NO_2_^−^), peroxynitrites, etc. These reactive species are known to be involved in a wide range of intracellular and intercellular processes^5^. Until now, major attention has been paid in plasma medicine to the monitoring of RONS induced in PAL used in indirect treatments^6^, and some works have investigated their storage by freezing the PAL but this is not always possible^7^. However, transportation and diffusion from suitable biomaterials of these RONS for *in situ* therapy remains to be explored.

Hydrogels can be an asset for that aim, as they have characteristics such as biocompatibility, *in vivo* biodegradability and ductility that are key features in the design of advanced biomaterials^8,9^. These highly swollen 3D networks of macromolecules have emerged as powerful candidate biomaterials for the local delivery of a variety of drugs at physiologically relevant doses for prolonged periods of time. Our hypothesis is that due to their high water contents and porous network they could be suitably used as carrier for RONS generated in plasma-activated liquid, providing new alternatives for therapies based on cold plasmas. Hydrogels can be based on natural polymers (e.g. polysaccharides, gelatine and fibrin), synthetic polymers (e.g. ethylene oxide, vinyl alcohol or acrylic acid) or semi-synthetic polymers (mixture between both natural and synthetic polymers)^10^. Hydrogels obtained from natural polymers have many advantages including low toxicity and good biocompatibility^11^. Alginate used in this work is obtained from brown algae and is typically used to produce hydrogels for a variety of applications in drug delivery and tissue engineering^12^. Alginate hydrogels can be prepared by simple gelation with divalent cations such as Ca^2+^ ^13,14^ so can be easily formed *in situ* in the body in contact with body fluids or in the lab in contact with Ca^2+^ containing solutions.

The purpose of this work is to evaluate the potential of employing alginate-based hydrogels as vehicles of RONS generated by atmospheric plasmas. Specifically, we analyse whether there are any chemical modifications in the structure of the alginate and its hydrogel-forming ability. In views of their possible therapeutic applications, their biological properties are investigated: biocompatibility of the plasma-treated polymer and cytotoxicity of the RONS generated therein.

## Results

### CAP produces high amount of RONS in alginate

CAP treatments of several concentrations of alginate (between 0.2% and 2%) produced nitrites (NO_2_^−^) and hydrogen peroxides (H_2_O_2_) in all cases. However, plasma treatment of high concentrations of alginate revealed a hampered diffusion of the reactive species (Supplementary Fig. 1). Since this lack of homogeneity in the diffusion of the reagent influenced the precise detection of RONS, the solution of 0.5 wt% alginate was selected for further work.

**Figure 1:**
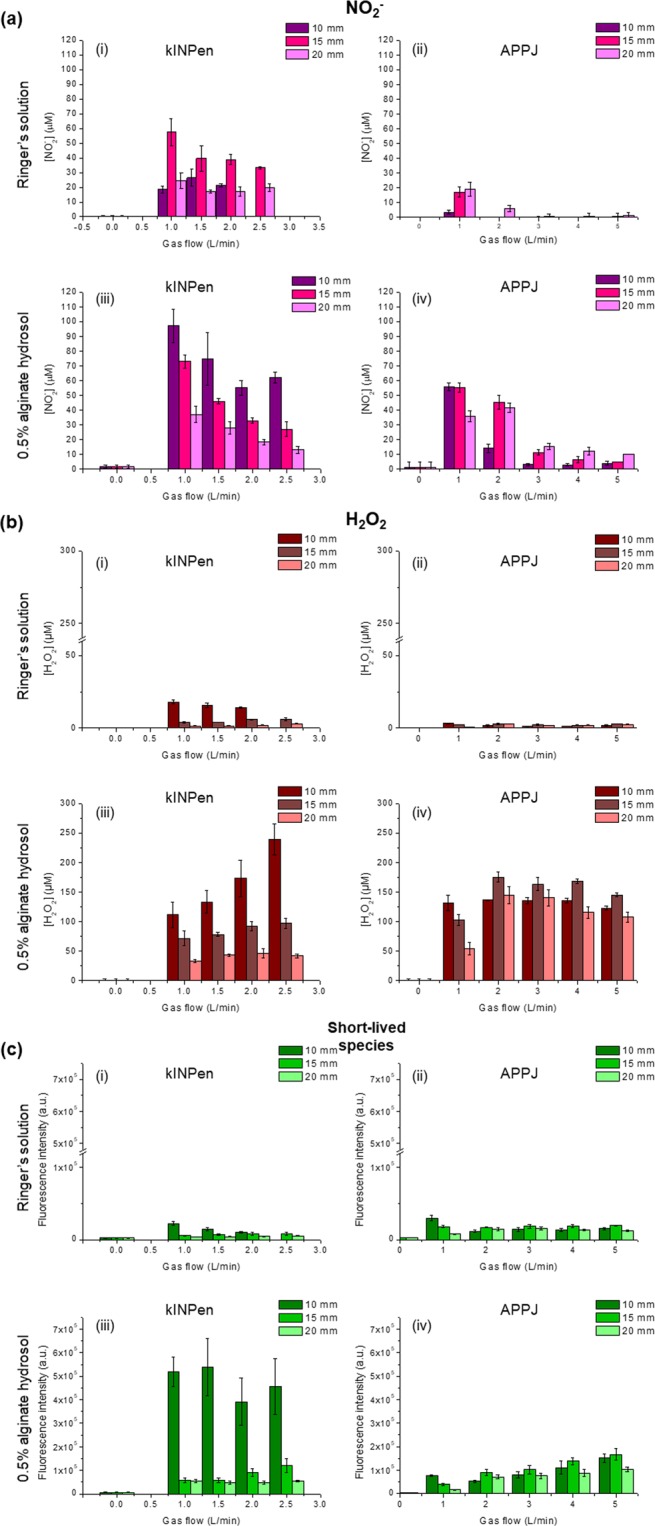
Influence of kINPen (left) or APPJ (right) distance to the sample and gas flow on the generation of NO_2_^−^ (**a**), H_2_O_2_ (**b**) and short-lived species (**c**) in Ringer’s saline and in 0.5% alginate solutions. Treatment time was fixed at 90 s.

Figure 1 presents the quantification of nitrites, hydrogen peroxide and short-lived species generated by plasma as a function of gas flow and distance to the sample in 0.5 wt% alginate or Ringer’s saline, used as control, for 90 s plasma treatment time.

Plasma treatment of alginate allowed much higher generation of RONS (NO_2_^−^, H_2_O_2_ and short-lived species) than those obtained in Ringer’s saline. In particular, the amounts of NO_2_^−^ generated by APPJ in Ringer at gas flows above 2 L/min are very low and barely visible (Fig. 1(a) (ii)). For both kINPen and APPJ-treated alginate, a higher loading of NO_2_^−^ was achieved for short distance to the sample and low gas flows (Fig. 1a). Higher concentrations of nitrites were generated using kINPen for all studied conditions.

The effects of plasma jet distance and gas flow on alginate followed a different trend when H_2_O_2_ was concerned (Fig. 1b). Increasing gas flow rates led to higher concentration of H_2_O_2_ in alginate, and short distances were still more suitable to generate higher amount of species. The amount of peroxides generated in Ringer’s was several-fold lower than in alginate, and the effects of gas flow or distance were minimized. kINPen at 10 mm distance reached the highest concentrations of H_2_O_2_, but longer distances produced less peroxides than APPJ. Regarding APPJ, similar H_2_O_2_ concentrations were obtained between 2 and 5 L/min disregard the distance between the liquid and the jet. The maximum amount of H_2_O_2_ in alginate generated by APPJ was obtained using 15 mm nozzle distance and gas flow between 2 and 4 L/min.

kINPen treatment of 0.5% alginate and Ringer’s saline highlighted that short distance enhances the generation of short-lived species (Fig. 1c). However, no significant differences were observed with the variation of gas flow for the alginate. The most efficient treatment of alginate with regard to the production of short-lived species was with kINPen at 10 mm distance and 1 L/min.

These results with plasma-treated alginate contrast with those obtained for plasma-activated Ringer’s solution in which APPJ treatment was more effective than kINPen in similar conditions. As observed with the long-lived species recorded (NO_2_^−^ and H_2_O_2_), the capacity for generating RONS was much higher in alginate than in plasma-activated Ringer’s saline, i.e. with up to 25 times higher concentrations of short-lived species for kINPen treatment of alginate with respect to physiological solution.

**Figure 2:**
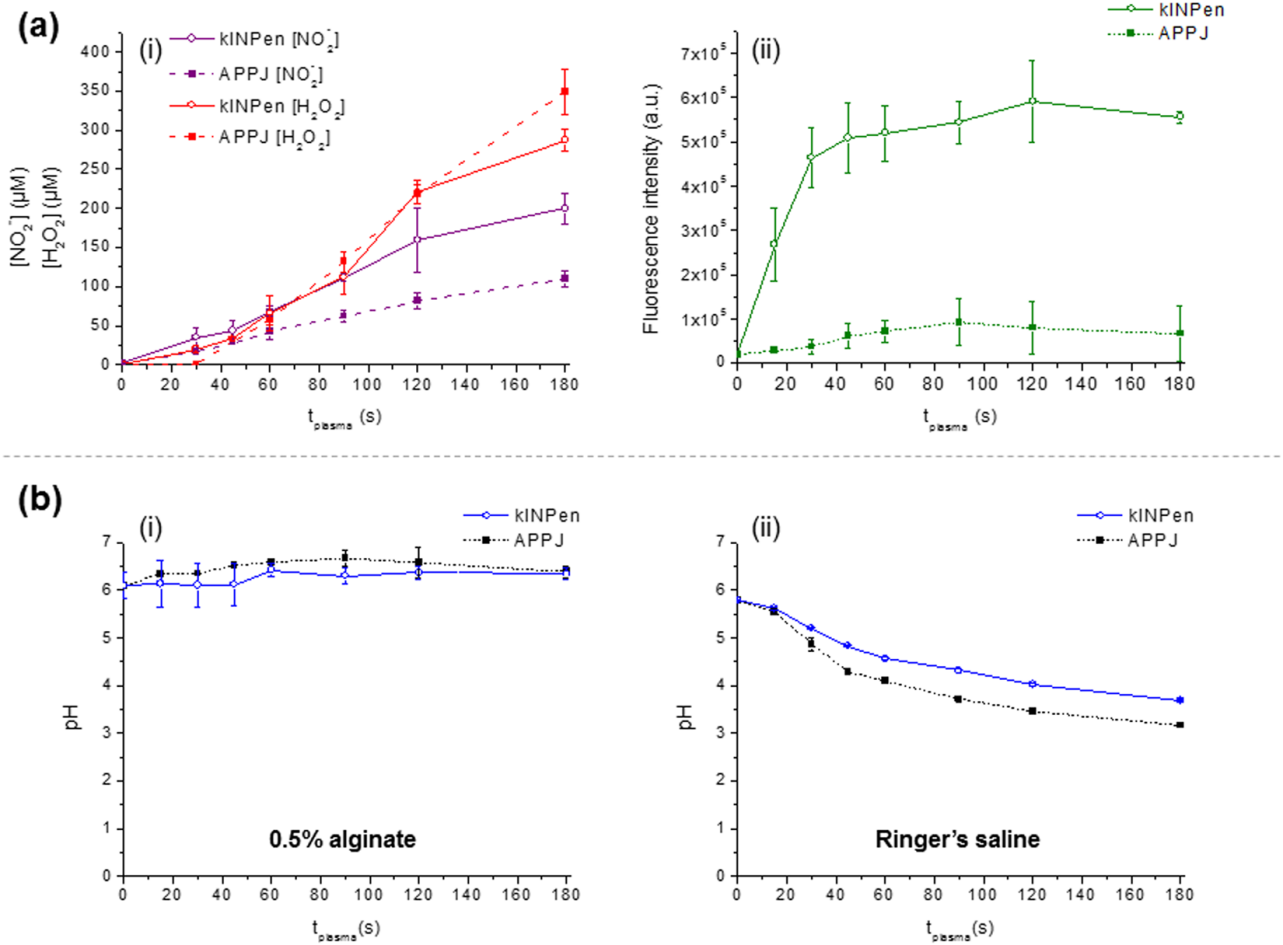
Influence of plasma treatment time on the generation of NO_2_^−^, H_2_O_2_ (i) and short-lived species (ii) in 0.5% alginate using kINPen or APPJ at 1 L/min and 10 mm distance (**a**). pH evolution as function of plasma treatment time of 0.5% alginate (i) and Ringer’s saline (ii) (**b**).

Plasma treatment time progressively increased the concentration of RONS ([NO_2_^−^], [H_2_O_2_] and short-lived species) in 0.5% alginate with both plasma jets (Fig. 2a). While the generation of H_2_O_2_ was relatively similar between kINPen and APPJ, it was observed that, independently of plasma treatment time, kINPen generated more NO_2_^−^ than APPJ. However, important differences were recorded among both sources with regard to generation of short-lived RONS: kINPen being much more effective than APPJ from short treatment times.

### CAP does not affect physic-chemical properties of alginate

**Figure 3:**
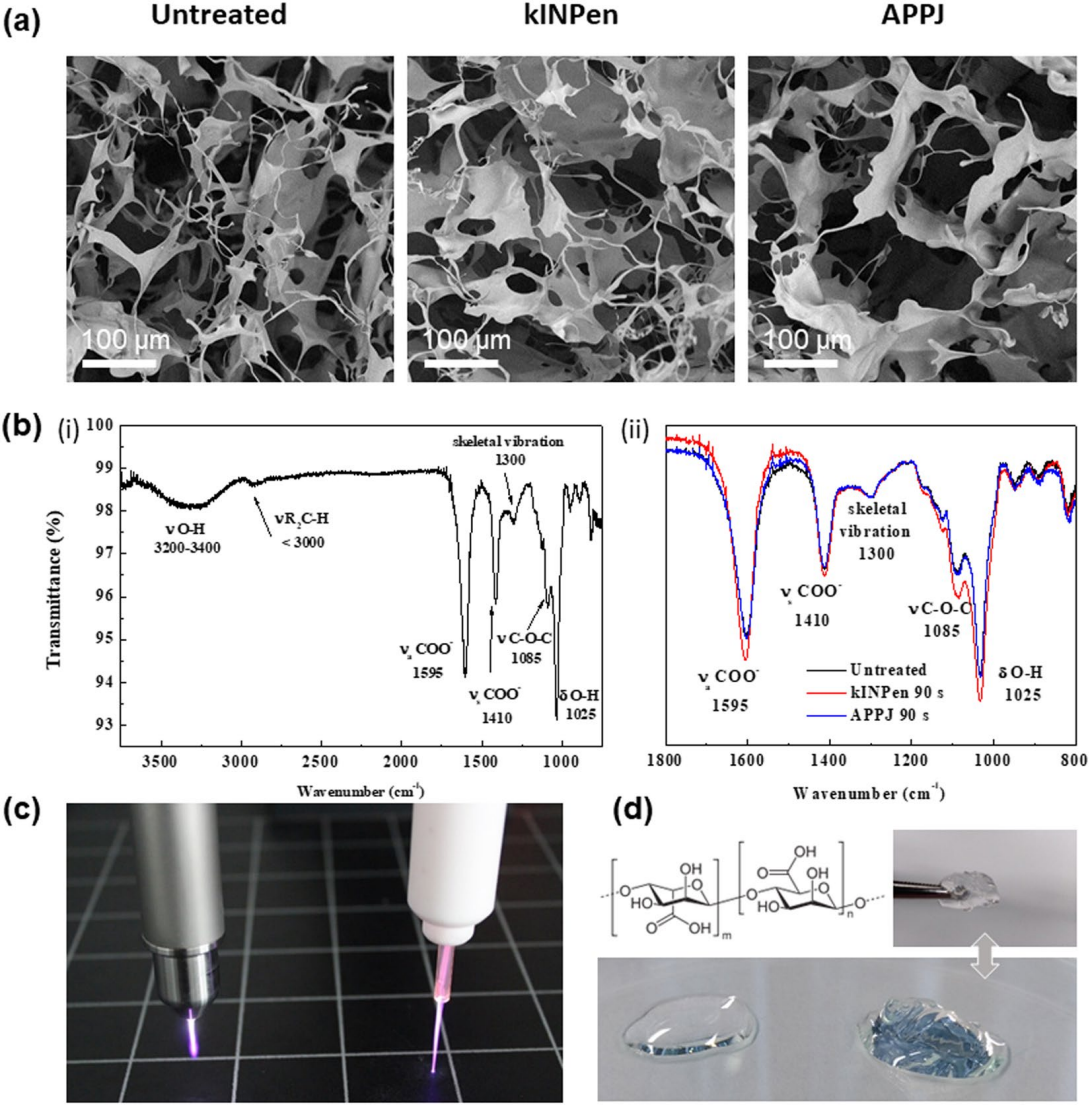
SEM micrographs (**a**) and FTIR-ATR spectra (**b**) of untreated (i), kINPen- and APPJ-treated 0.5% alginate for 90 s, at 10 mm distance and 1 L/min. Digital picture of kINPen and APPJ in operation (**c**). Chemical structure of alginate and digital pictures of the alginate solution (left side) and of the cross-linked alginate hydrogel (right side) (**d**).

In this section 2 mL of 0.5% alginate were treated with either kINPen or APPJ under selected conditions (10 mm, 1 L/min) (pictures shown in Fig. 3c). The pH of plasma-treated alginate with any of both plasma jets remained unchanged with treatment time (Fig. 2b). This contrasts to the pH of Ringer’s saline that decreased down to pH between 3.2 and 3.7 after 3 min of treatment.

Typical porous structures of lyophilized alginate (Fig. 3a) were observed by Scanning Electron Microscopy (SEM) and no differences were found after the plasma treatment. Similarly, Fourier Transform Infrared (FTIR-ATR) spectra of the 0.5% alginate (Fig. 3b) revealed no significant shifts between the different plasma jets studied (i.e., less than 5 cm^−1^). According to the literature, FTIR-ATR spectra of the sodium alginate presents seven characteristic bands from 2000 to 650 cm^−1^ region of the FTIR spectra^15,16^ which remain unaltered with plasma treatment either using kINPen or plasma needle, indicating that plasma jet treatment of the solution did neither affect the main chemical bonds of the polymer network nor the ability of the solution to form a hydrogel (Fig. 3d). Only a minor change in intensity is found in the broad −OH stretching band within the 3400–3200 cm^−1^ range (not shown). This peak corresponds to a convolution envelope including both FTIR bands from water molecules (usually appearing between 3700–3100 cm^−1^)^17^ and the bands that correspond to the hydroxyl groups from the hydrogel. Since this peak can be highly dependent on the freeze-drying process of the samples, this hampers drawing conclusions from the peaks appearing in this range.

### Crosslinking is a critical step

Since our final aim was to evaluate the ability of hydrogels to act as reservoirs for RONS generated by plasma, crosslinking of the alginate was an essential step. Thus, the 0.5% alginate solutions were plasma-treated, cross-linked for 5 min in a CaCl_2_ solution, and then rinsed to eliminate excess of crosslinker that could be toxic for further cell testing.

**Figure 4:**
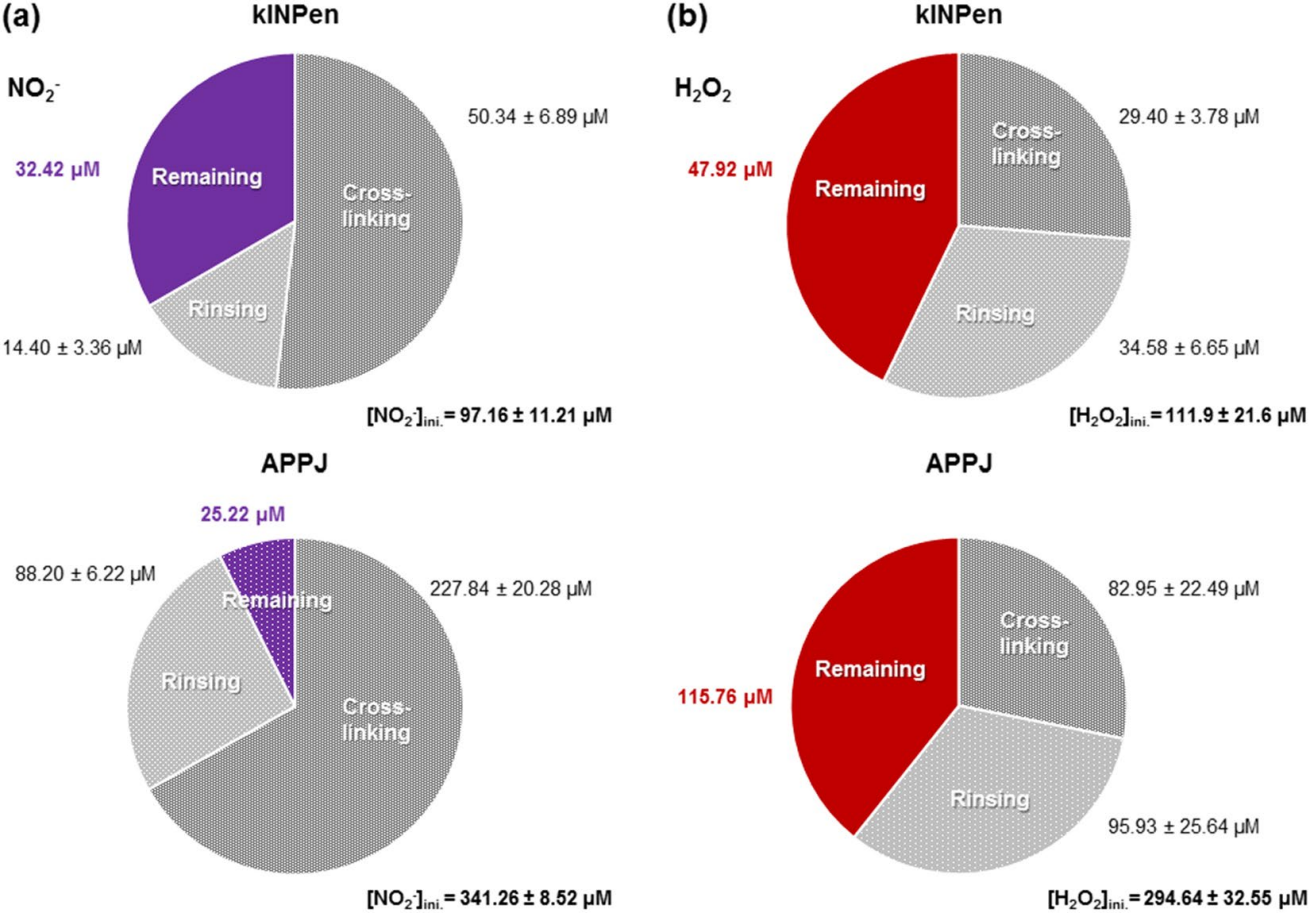
Total concentration of NO_2_^−^ (**a**) and H_2_O_2_ (**b**) released during crosslinking and rinsing processes of the alginate hydrogel previously treated by kINPen for 90 s and APPJ for 15 min (10 mm, 1 L/min). The proportion of RONS remaining in the hydrogel after crosslinking and rinsing are highlighted in violet for NO_2_^−^ and red for H_2_O_2_.

A significant part of the RONS generated by plasma in the alginate was released during the crosslinking process (Fig. 4). Most NO_2_^−^ was lost from plasma-treated alginate during its crosslinking (51.8% for kINPen and 66.7% for APPJ) and the rinsing process (14.8% and 25.8%, respectively). Thus, these steps left the alginate hydrogel with only 33.4% for kINPen and 7.5% for APPJ of the initial NO_2_^−^ plasma-loading, which corresponds to 32.4 µM and 25.2 µM respectively.

**Figure 5:**
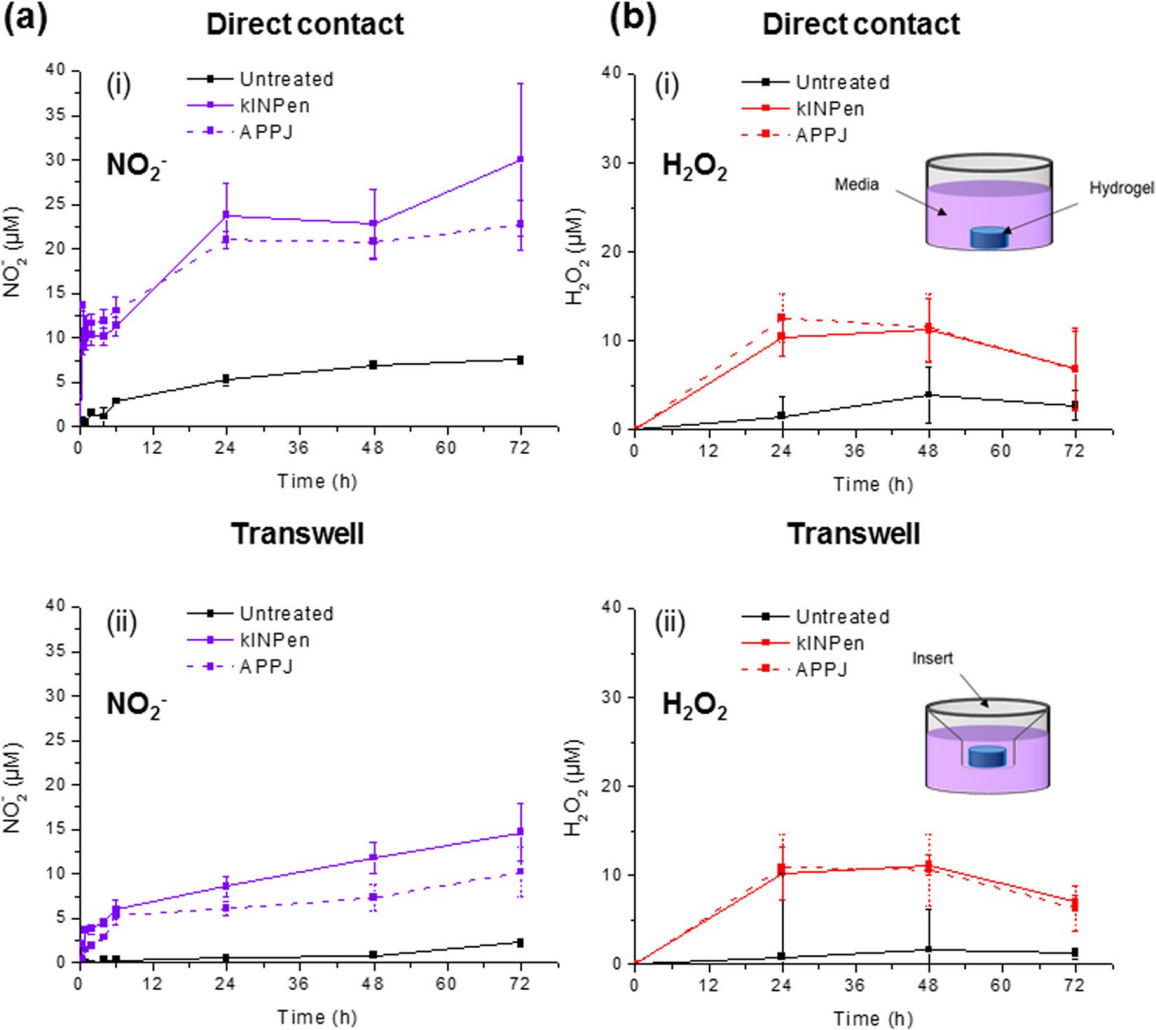
Cumulative release profiles of NO_2_^−^ (**a**) and H_2_O_2_ (**b**) from the RONS-loaded 0.5% alginate hydrogels to cell culture media. The alginate hydrogels had been treated by kINPen or APPJ for 90 s or 15 min, respectively (at 10 mm, 1 L/min), crosslinked and rinsed. Release was evaluated either in direct contact (i) or in Transwell (ii).

In the case of H_2_O_2_, a lower fraction was lost during the whole crosslinking process (crosslinking + rinsing). A final percentage of 42.8% and 39.3% (in kINPen- and APPJ-treated alginate, respectively) remained in the hydrogel with respect to the initial loading, corresponding to final amounts of peroxides of 47.9 μM and 115.8 μM. These can be considered as the initial amount of nitrites and peroxides loaded in the alginate hydrogel at the beginning of the release experiments presented in Fig. 5.

### CAP-treated hydrogels are not cytotoxic

The diffusion of species remaining in the plasma-treated alginate hydrogels after the crosslinking process to the cell culture medium (Fig. 5) was evaluated either in Transwell experiments or in direct contact with the medium.

Figures 5a,b show the respective NO_2_^−^ and H_2_O_2_ release kinetics from plasma-treated alginate hydrogel to the cell culture medium during 72 h, using the hydrogel in direct immersion (i) or using an insert (Transwell experiments) (ii). Direct immersion of the alginate hydrogel led to higher release amount of NO_2_^−^ from the hydrogel than Transwell experiments. Plasma-treated hydrogel presented a maximum release of nitrites of 30.01 ± 8.05 µM for kINPen and 22.68 ± 2.77 µM for APPJ after 72 hours for direct immersion of the hydrogel, which corresponds to a 92.6% and 89.9% of the initial loading of NO_2_^−^.

Half NO_2_^−^ release was recorded (14.65 ± 3.29 µM and 10.19 ± 2.75 µM, respectively) when placing the hydrogel in an insert (this corresponds to 45.2% and a 40.4% of the initial concentration of nitrites in the hydrogel), in kINPen and in APPJ respectively.

Regarding H_2_O_2_ release from the plasma-treated alginate (Fig. 5b), low amount of hydrogen peroxides was observed either in Transwell or in direct contact, with higher values of H_2_O_2_ in the release media around 10 µM.

**Figure 6:**
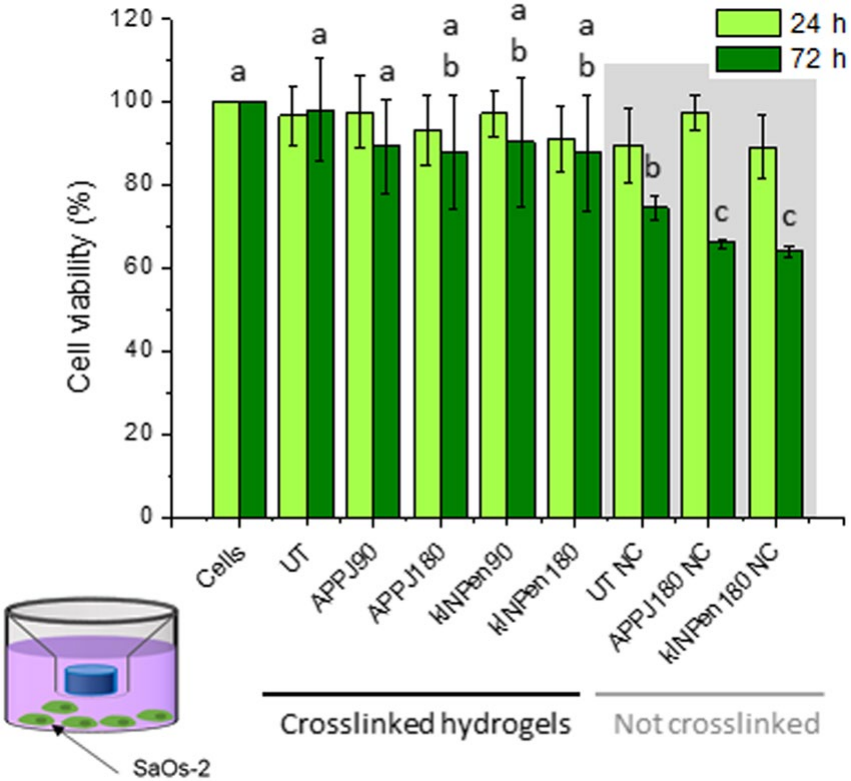
SaOS-2 cell viability of untreated (UT), APPJ- and kINPen-treated alginate hydrogels for 24- and 72-hour Transwell cell culture at treatment times of 90 and 180 s. Cell viability using non-crosslinked alginate solutions (NC) is presented on grey background. a,b,c indicate statistically significant differences.

The effect of the release of RONS from the plasma-treated alginate was studied on Sarcoma osteogenic cells (SaOS-2) and results are presented in Fig. 6. No significant differences were observed in SaOs-2 cell viability between untreated and plasma-treated crosslinked hydrogels placed in Transwell with respect to control, either for 24 or 72 hours. After crosslinking the hydrogels had undergone washing process, so few RONS remained in the material. Therefore, the lack of toxicity in the crosslinked hydrogels indicates that the plasma treatment of the alginate did not induce cytotoxic alterations in the alginate chains. In contrast, non-crosslinked alginate solutions presented a decrease of cell viability after 72 hours with respect to control, with cell viability of 76.0% for UT NC and 65.9% and 63.9% for APPJ180 NC and kINPen180 NC, respectively.

## Discussion

In this work alginate hydrogels have been shown to be suitable vehicles for RONS produced by atmospheric pressure plasma jets. It is widely admitted that the CAP-generated RONS are basic anti-cancer factors supressing cancer cell proliferation in *in vitro* cell cultures^18–23^ and are also essential in the treatment of chronic wounds^24^. Here, two atmospheric pressure plasma jets are compared: a single-electrode jet working with helium and the kINPen, a widely extended plasma jet working with argon. The rationale for selecting these two jets with different characteristics is to allow for easier extrapolation of the results.

It is known that a variety of RONS can be formed by CAP in water or saline solutions such as Phosphate Buffer Saline (PBS)^25–29^ and, as recorded here, in Ringer’s saline. Many parameters affect the generation of RONS in solutions, the differences in chemical composition of the treated solution being critical to that aim^6^. Three species were quantified here: H_2_O_2_, NO_2_^−^ and long-lived species (Fig. 1(a–c) (i, ii)), their concentration increasing with treatment time (Fig. 2a) with both plasma jets, APPJ and kINPen. As reviewed by Jablonowski *et al*.^24^, RONS in the liquid phase can either be generated by direct interaction at the plasma/liquid interface^21,30^, via transfer of the reactive species from the gas phase into the liquid^31,32^, or by secondary or tertiary reactions in the bulk liquid^2^.

It can be observed that the concentration of RONS generated in alginate was several-fold higher than in Ringer’s saline, ranging between 2 (NO_2_^−^), 10 (H_2_O_2_) and 25 (short-lived RONS) -fold higher for kINPen and slightly lower for the He APPJ needle. This can be explained by the following: Nitrites are formed in plasma treated liquids through the dissolution of nitrogen oxides formed in the gas phase of the plasma jet. In general, in acidic conditions, nitrous acid (which is one of the major sources of NO_2_^−^) is not stable and decomposes into nitrogen dioxide, which may react with OH^•^ to form peroxinitrous acid.

Another reaction that is promoted in acidic solution takes place between NO_2_^−^ and H_2_O_2_ (reaction 1) and is another source of peroxynitrites^6,33–35^.

1 $${{\rm{NO}}}_{2}^{-}+{{\rm{H}}}_{2}{{\rm{O}}}_{2}+{{\rm{H}}}^{+}\to {\rm{ONOOH}}+{{\rm{H}}}_{2}{\rm{O}}$$

Peroxinitrous acid is not stable at acidic pH and converts to NO_3_^−^, as shown by Bruggeman *et al*.^30^.

As Ringer’s saline is progressively acidified by the CAP treatment (Fig. 2b) - due to the formation of nitrites - these reactions can take place and decrease the amount of H_2_O_2_ and NO_2_^−^ in the liquid (Fig. 1). On the contrary, the close to neutral pH found in the alginate solutions (Fig. 2b), avoids this reaction taking place and may allow for the accumulation of much higher concentrations of these RONS. This hypothesis is confirmed by CAP treatment of a buffered solution (PBS) at the same pH of 6.5 as alginate (Supplementary Fig. 2). It can be observed that the concentrations of RONS generated by CAP treatment in PBS are similar with those obtained in alginate.

The screening of the plasma treatment conditions pointed out that the amount of the different reactive species (NO_2_^−^, H_2_O_2_ and short-lived species) can be easily tuned, a crucial feature to be able to control the dose of RONS. For instance, as observed in other works^36^, on increasing the gas flow rate, after a certain point the concentration of NO_2_^−^ decreases for both plasma jets (Fig. 1a). This can possibly be ascribed to the fluid dynamics in the gas phase which play a major role in the species that can reach the liquid surface and thus, on the generation of RONS in liquids. It has been reported that at low flow rates the jet effluent follows a laminar mode that allows the mixing of air with the noble gas generating the discharge (and thus generation of more RONS in the gas phase)^1,21,26,35^. Contrarily, at higher gas flows, the effluent enters a turbulent mode that impairs mixing of air and decreases the amount of certain RONS generated^21^. For a deeper understanding of the reactions taking place in liquids, Verlackt *et al*.^26^, presented a 2D fluid dynamics model for the interaction between kINPen and a liquid (water). Despite the 0.5% alginate being more viscous than water, the plasma gas phase fluid dynamics may be extrapolated and the same descriptions valid at this point, since to our knowledge no modelling with hydrogels has been reported until now.

Alginate solutions are studied in this work given their ability to crosslink *in vivo* with Ca^2+^ ions from the body and form stable hydrogels which can allow drug delivery of RONS with a minimally invasive strategy. The higher concentrations of RONS generated by CAP in alginate, which displayed no apparent changes in the structure of the polymer (Fig. 3) were progressively released to a surrounding medium (Fig. 5). However, to simulate the crosslinking in the laboratory and obtaining the hydrogel, it is necessary to add a CaCl_2_ solution to the alginate solution. This leads to an important loss of the initial amount of RONS by diffusion to the crosslinking solution (Fig. 4). Although the loss of RONS in the crosslinking process could partly be limited during crosslinking by reducing the volume and increasing the concentration of CaCl_2_ of the crosslinking solution, this leak of RONS is inevitable if working *in vitro*. The final values of RONS in the final hydrogel are between 7% and 34% of the initial amounts generated by CAP in the alginate hydrosol, depending on the reactive species and the initial plasma treatment performed. In particular, the final amount remaining in the alginate hydrogel for further release of NO_2_^−^ was 32.4 µM for 90 s kINPen treatment and 25.2 µM for 15 min APPJ treatment (10 mm, 1 L/min); concentration of H_2_O_2_ was of 47.9 µM and 115.8 µM, respectively. These amounts of RONS are still higher in hydrogel compared to Ringer’s saline (Fig. 1), which is an advantage to the use of alginate with respect to saline solution. Furthermore, for *in vivo* application, as crosslinking would take place in the body with the Ca^2+^ in body fluids, all RONS would be available for local delivery.

Release experiments showed final nitrite release of 30.0 µM for kINPen and 22.7 µM for APPJ-treated alginate hydrogel after 72 h, in direct contact with cell culture medium, corresponding to release percentages of 92.4% and 89.9% of the initial concentration, respectively. The final NO_2_^−^ concentrations go down to 14.7 µM and 10.2 µM, respectively, when the plasma-treated hydrogels were placed in suspension in the cell culture media using an insert (same configuration between the release and cytotoxicity experiments). Regarding H_2_O_2_, the highest amount (~10 μM) was obtained after 24 h of release for both plasma devices. A decreasing trend of H_2_O_2_ concentration was observed in the release media from 48 to 72 hours, that may be attributed to an ageing of H_2_O_2_, as observed in cell culture media^37^.

The viability of SaOS-2 was not affected when cells were cultured in indirect contact with untreated and plasma-treated crosslinked hydrogels for both kinds of plasma treatments (Fig. 6). Despite the high loading of RONS in the alginate solutions achieved by plasma treatment, the low amount of RONS remaining after crosslinking (Figs 4 and 5) does not affect cell viability, which agrees with previous works^6,38^. As most RONS were washed away by the crosslinking + washing process, this allowed to ascertain that the biomaterial itself remains fully biocompatible after the plasma treatment (no significant differences were observed in crosslinked alginate between treatments or with respect to controls, Fig. 6).

To evaluate the biological efficacy of the plasma-generated RONS within the alginate, non crosslinked (NC) polymer solutions were employed (Fig. 6). A slight decrease in cell viability was observed for the untreated alginate solution (UT NC) that could be attributed to calcium sequestration from the cell culture media by the alginate. The plasma-generated RONS lead to a further decrease of cell viability from the untreated (UT NC) to the plasma-treated alginate solutions. Cell viability after 72 hours decreased to 65.9% for APPJ180 NC and 63.9% for kINPen180 NC, respectively. This decrease on SaOS-2 cell viability can be ascribed to the concentration of H_2_O_2_ released to the media from the plasma-treated alginate solutions, and available for interaction with cells was of 39.79 ± 3.49 μM for APPJ180 NC and 37.68 ± 2.96 μM for kINPen180 NC.

The decrease in cell viability obtained with these levels of RONS is in line with previous works^38^, where APPJ plasma treatment of cell culture medium resulted in a progressive decrease of the viability of SaOS-2 with the increase of RONS concentration in culture media. In that work, cell viability was found between 92 and 20% for APPJ treatment times of 10–30 min. The higher cytotoxicity in that work^38^ has to be directly related with the higher amount of RONS present in the McCoy’s cell culture medium (ie. H_2_O_2_: 98 μM for 10 min APPJ treatment to 290 μM for 30 min APPJ treatment) with respect to the amounts found here. Despite here only NO_2_^−^, H_2_O_2_ and short-lived ROS were detected, many studies have described the anti-carcinogenic effects of plasma by many other reactive species such as O_2_^−^, OH^*^, NO, O, NO_3_^−^, NO_2_^−^ and ONOO^−^ ^21^, and other works^25,39^ investigated artificially supplementing liquids with the same concentrations of H_2_O_2_ and/or NO_2_^−^ and did not observe the same effects as with plasma, confirming the hypothesis that the complex of RONS generated by plasma is necessary for its biological action.

Thus, generating reactive species by cold atmospheric plasmas in alginate-based biomaterials and their release opens great perspectives in the design of new implantable biomaterials for plasma therapies. These results have important implications in many biomedical applications of alginate hydrogels, including tissue engineering and drug delivery.

## Methods

### Materials

Sodium alginate (Na-alginate MW: 10000–600000 g/mol) in powder form was purchased from Pancreac. Potassium chloride (KCl, Panreac), sodium chloride (NaCl, Sigma-Aldrich) and calcium chloride dehydrate (CaCl_2_.2H_2_O, Sigma-Aldrich) were used for the preparation of Ringer's saline solution. Phosphoric acid (85%; MW: 98 g/mol, Panreac), sulphanilamide (M.W: 172.20 g/mol, Sigma-Aldrich) and N-(1-naphtyl)ethylenediamine (NEED, MW: 172.20 g/mol, Sigma-Aldrich) were used for synthesis of Griess reagent. Calcium chloride (CaCl_2_, 96% anhydrous, MW: 110, 98 g/mol), sodium nitrite (NaNO_2_ MW: 69 g/mol) and sodium azide (NaN_3_, MW: 65 g/mol), in powder form, were obtained from Sigma-Aldrich. Titanyl oxysulphate (TiOSO_4_, MW: 159.90 g/mol; 27–31%_wt_ in H_2_SO_4_), hydrogen peroxide (H_2_O_2_, MW: 34.01 g/mol; 30%_wt/wt_ in H_2_O), peroxidase from Horseradish type VI (HRP) (250 U/mg, Sigma-Aldrich) and 2′,7′-Dichlorofluorescein diacetate (DCFH-DA) (≥97%) were purchased from Sigma-Aldrich. Amplex Red reagent was provided by Invitrogen (ref. A12222). Helium and argon gas were provided by Praxair, Spain.

Sarcoma osteogenic cells (SaOs-2, ATCC, USA) were expanded in McCoy’s 5A culture medium (Sigma Aldrich). Foetal Bovine Serum (FBS) and Penicillin/Streptomycin (P/S) (50 U/ml and 50 μg/ml, respectively) were purchased from Invitrogen. Cells from passage between 24 and 32 were used in all experiments. Cell Proliferation Reagent WST-1 used for cell viability determination was purchased from Roche Diagnostics GmbH (ref. 05015944001).

### Preparation of alginate solutions and hydrogels

The alginate solutions were obtained by mixing the dry sodium-alginate powder with DI water in a SpeedMixer (DAC 150.1 FVZ-k, 3500 rpm) for 15 min at 0.5% w/w. Alginate solutions (or hydrosols), that refer to the physical state of the alginate before crosslinking of the polymer chains^40^, were stored at 4 C and used within a lifespan of 2 weeks. The alginate hydrogels were obtained by ionic crosslinking of the hydrosol using a 150 µL of a 50 mM calcium chloride (CaCl_2_) solution for 200 µL of alginate hydrosol for 5 min. Subsequent rinsing using 100 µL of DI water of the formed hydrogel was performed for 5 minutes before its use for release and cell culture experiments, to eliminate the excess of calcium coming from the crosslinking solution. Crosslinking and rinsing solution used for obtaining the hydrogel were kept for determination of [NO_2_^−^] and [H_2_O_2_], and the formed hydrogels were used for 72-hour release experiments. For cell experiments using alginate hydrogels, all the processes leading to the preparation of the formed hydrogel were carried out under sterile conditions. Alginate powder was sterilized by low-pressure plasma treatment using a low-pressure radio-frequency plasma.

### Plasma treatments

In this study, two kinds of atmospheric plasma jet were used: a commercially available cold atmospheric plasma jet kINPen IND (NEOPLAS Tools, Germany)^6^, operating with argon and an atmospheric pressure plasma jet (APPJ) using He as plasma gas in a jet design based on a single electrode as described elsewhere^41^ (Fig. 3c). Gas flow was regulated between 1 and 2.5 L/min for kINPen and between 1 and 5 L/min for APPJ by using Ar and He Bronkhorst Mass View flow controllers (BRONKHORST, Netherlands), respectively.

All plasma treatments of alginate for RONS quantification were performed on 200 µL of the alginate solution (before crosslinking) in 96-well plates, with adistance between the nozzle and the sample surface between 10 and 20 mm.

### Detection of RONS in alginate hydrosols

Determination of NO_2_^−^ concentration in plasma-treated alginate hydrosol was performed using Griess reagent^30,35,42,43^. The Griess reagent used was obtained by dissolving 1%_wt/v_ of sulphanilamide, 0.1%_wt/v_ of NEED and 5%_w/v_ of phosphoric acid in de-ionized water. 200 μL of Griess reagent were added on 200 μL of sample in 96 well-plates. The plates were incubated for 10 min at room temperature protected from the light. The absorbance was measured at λ_abs_ = 540 nm using a Synergy HTX Hybrid Multi Mode Microplate Reader (BioTek Instruments, Inc., USA). The [NO_2_^−^] in each sample was determined from the absorbance values by using a calibration curve made from NaNO_2_ dilutions in alginate hydrosols.

**Figure 7:**
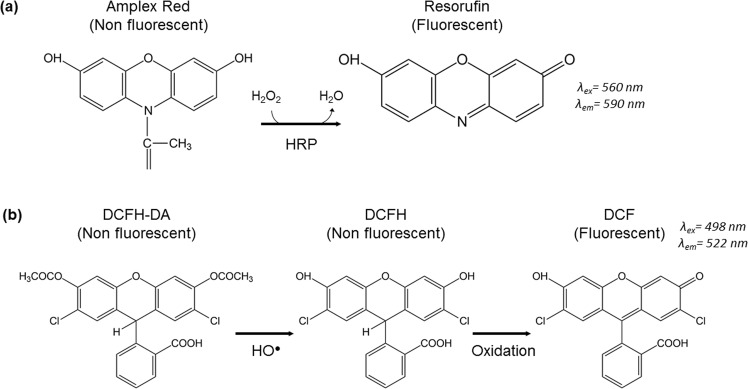
Chemical reactions involved in the fluorescent probes used for the detection of H_2_O_2_ (**a**) and short-lived reactive species (**b**) in alginate hydrosol.

The concentration of hydrogen peroxide was determined by reaction of H_2_O_2_ with Amplex Red in presence of HRP enzyme that leads to the creation of resorufin, a fluorescent product (Fig. 7a). Amplex Red/HRP reagent consists in 100 µM of Amplex Red and 0.25 U/mL HRP in DI water. Since the higher concentration of H_2_O_2_ able to be processed properly by this reagent is around 10 µM of H_2_O_2_, plasma-treated alginate hydrosols were diluted 200 times previously to the addition of the reagent. In this case, for hydrogen peroxide detection, 50 μL of the Amplex Red/HRP reagent was added to 200 μL of the 200×-diluted alginate sample in a 96-well plate and incubated for 30 min at 37 °C. Subsequent fluorescence measurements were performed by means of a Synergy HTX Hybrid Multi Mode Microplate Reader (BioTek Instruments, Inc., USA), with fluorescence filters centred at λ_ex_ = 560/20 nm and λ_em_ = 590/20 nm as excitation and emission wavelengths, respectively. Concentrations of H_2_O_2_ in alginate hydrosol generated by plasma treatment were obtained from the fluorescence values using a calibration curve made from 30% hydrogen peroxide solution in alginate.

Presence of short-lived RONS was determined *in situ* in plasma-treated 0.5% alginate solution using 2′,7′ Dichlorodihydrofluorescein diacetate (DCFH-DA), which is a scavenger of short-lived RONS^44^. DCFH-DA is a non-fluorescent dye which is hydrolyzed into its polar, but non-fluorescent form DCFH on the action of HO^•^ radicals. Oxidation of DCFH by the action of reactive oxygen species (ROS) turns the molecule into its highly fluorescent form 2,7-Dichlorofluorescein (DCF) that can be detected by fluorescence^42^ (Fig. 7b). Since 2′,7′-DCFH is a non-specific probe and can react with various short-lived species, such as OH^•^, HOO^•^, NO^•^, H_2_O_2_, ONOO^•^^35,45,46^, and due to the short lifespan of these species, no calibration curve can be done and results are expressed in fluorescence intensity. DCFH was previously incorporated to alginate hydrosol before plasma treatments in proportion 1 μL of 2 mM 2,7-DCHF for 150 μL of alginate hydrosol. 200 µL of the alginate sample containing DCHF were placed in 96-well black plate for plasma treatment. After 30-min incubation at room temperature, fluorescence intensity was read with a Synergy HTX Hybrid Multi Mode Microplate Reader using λ_ex_ = 485/20 nm and λ_em_ = 528/20 nm as excitation and emission wavelength filters, respectively.

As control, 0.9% Ringer’s solution was used. performed in the same treatment conditions as alginate hydrosol. Ringer’s saline was prepared by dissolving 8.60 g/L NaCl, 0.30 g/L KCl and 0.33 g/L CaCl_2_.2H_2_O in DI water, filtered by using 0.22-µm pore size MILLEXGP filter unit (Merck Millipore Ltd., Ireland).

### pH monitoring

2 mL of 0.5% alginate were placed in a 24 well-plates and treated using kINPen and APPJ (10 mm, 1 L/min). pH was measured by using a PC80 Multiparameter instrument (XS Instruments, Italy) with a Crison 50 14 electrode (Crison, Spain).

### FTIR-ATR

FTIR-ATR spectra of freeze-dried alginate hydrogels were recorded using a Nicolet 6700 spectrometer (Thermo Scientific), equipped with a Universal ATR sampling device with a germanium crystal. Spectra were acquired at room temperature in transmission mode as a function of the λ ranged between 4 000 and 675 cm^−1^ with 64 scans at a resolution of 1 cm^−1^. A background spectrum of air was scanned under the same instrumental conditions before each series of measurements.

### SEM

Lyophilized 0.5% alginate hydrosols were C-coated using an EMITECH K950X Turbo Evaporator (Quorum Technologies Ltd., UK). All samples were imaged in a Phenom XL SEM (Phenom-World B.V., The Netherlands) under high vacuum at 5 kV and a 5 mm working distance.

### Release of RONS

200 µL of 0.5% alginate hydrosol in 96-well plate were treated by kINPen for 90 s, 10 mm and 1 L/min and APPJ for 15 min, 10 mm and 1 L/min. Since lower amount of NO_2_^−^ are generated in alginate with APPJ than kINPen, a longer plasma treatment time for APPJ has been performed to be able to reasonably detect [NO_2_^−^] with absorbance values within the plate-reader working range. 200 µL of untreated and plasma-treated hydrosols were cross-linked in a 96-well plate by using 50 mM CaCl_2_ solution for 5 min. Afterward, formed alginate hydrogels were transferred to another well with 100 µL distilled water for rinsing of the excess of calcium chloride solution for 5 min. Plasma treatments and crosslinking process for RONS release experiments were carried out in the same conditions used for cell culture experiments to be able to relate the release of RONS from the alginate hydrosol with the biological effects.

Formed alginate hydrogels were transferred to CORNING Transwell polyester membrane cell culture insert (Sigma-Aldrich), with a 6.5 mm diameter and a 0.4 µm pore size and placed in suspension in 1 mL volume of cell culture media in 24-well plates. For the monitoring of the release kinetics of RONS from the alginate hydrogels 100 µL of the cell culture medium used as release media were withdrawn at determined time points for subsequent quantification of NO_2_^−^ and H_2_O_2_. 100 µL of fresh medium was replaced after each sample collection. Final volumes of release media have been measured at the end of release experiment to take into account the volume correction in the concentration calculations of NO_2_^−^ and H_2_O_2_.NO_2_^−^ and H_2_O_2_ were quantified as described in the previous section.

### *In vitro* cell experiments

#### Cell culture

Sarcoma Osteogenic (SaOs-2) were used to study the cytotoxicity of the alginate hydrogels. The cell culture medium consisted of McCoy’s 5A with 10% FBS and 1% P/S. Cells were grown in 75 cm^2^ cell culture flasks at 37 °C in a 5% CO_2_ incubator and upon reaching 80% confluence. SaOs-2 were detached from the flask using trypsin (Invitrogen, Thermofisher) and 10000 cells/well were seeded into 24-well plates with 1 mL volume of culture medium. After 6 h-adhesion, plasma-treated sterile alginate hydrogels, previously prepared in sterile conditions, were introduced into a CORNING Transwell polyester membrane cell culture insert and placed in suspension in the well, to evaluate the effect of kINPen and APPJ plasma treatment of the alginate hydrogels on the SaOs-2 cell viability. As positive control, the same number of cells was placed without adding alginate. The cells were grown at 37 °C in a 5% CO_2_ incubator for another 72 h.

#### Cell viability

Influence of plasma-treated 0.5% alginate hydrogels on SaOs-2 cell viability was evaluated for kINPen and APPJ (10 mm, 1 L/min) for 90 and 180 s of plasma treatments. Plasma-treated alginate solutions were also studied for 180 s APPJ and kINPen plasma treatment. Cell viability was evaluated at 0, 24 and 72 hours. Cell culture media was replaced by preparation consisting of 250 µL of Cell Proliferation Reagent WST-1 in Mc Coy’s culture medium (1:10) and incubated for 1 hour at 37 °C. Afterward, 100 µL of the supernatant were transferred to another well for absorbance measurement at 440 nm. To evaluate the effects untreated and plasma-treated alginate hydrogels on SaOs-2 cell viability, normalization of the values was made with respect to the well containing cells only.

## Supplementary information

Supplementary Information
