# Supplementary material for: Production of reactive species in alginate hydrogels for cold atmospheric plasma-based therapies
Source: Sci Rep. 2019 Nov 6;9:16160. doi: 10.1038/s41598-019-52673-w (PMC6834627; doi:10.1038/s41598-019-52673-w)
Supplement: Supplementary file 1 — Supplementary Information [file 41598_2019_52673_MOESM1_ESM.docx]

**Production of reactive species in alginate hydrogels for cold atmospheric plasma-based therapies**

**Cédric Labay**1,2**, Inès Hamouda**1,2**, Francesco Tampieri**1,2**, Maria-Pau Ginebra**1,2,3**, and Cristina Canal**1,2

1Biomaterials, Biomechanics and Tissue Engineering Group, Dpt. Materials Science and Metallurgy, Universitat Politècnica de Catalunya (UPC), Escola d’Enginyeria Barcelona Est (EEBE), c/ Eduard Maristany 14, 08019 Barcelona, Spain

2 Barcelona Research Center in Multiscale Science and Engineering, UPC, Spain

3 Institute for Bioengineering of Catalonia (IBEC), Barcelona Institute of Science and Technology (BIST), c/ Baldiri i Reixach 10-12, 08028 Barcelona, Spain

*cristina.canal@upc.edu

**Supplementary information**


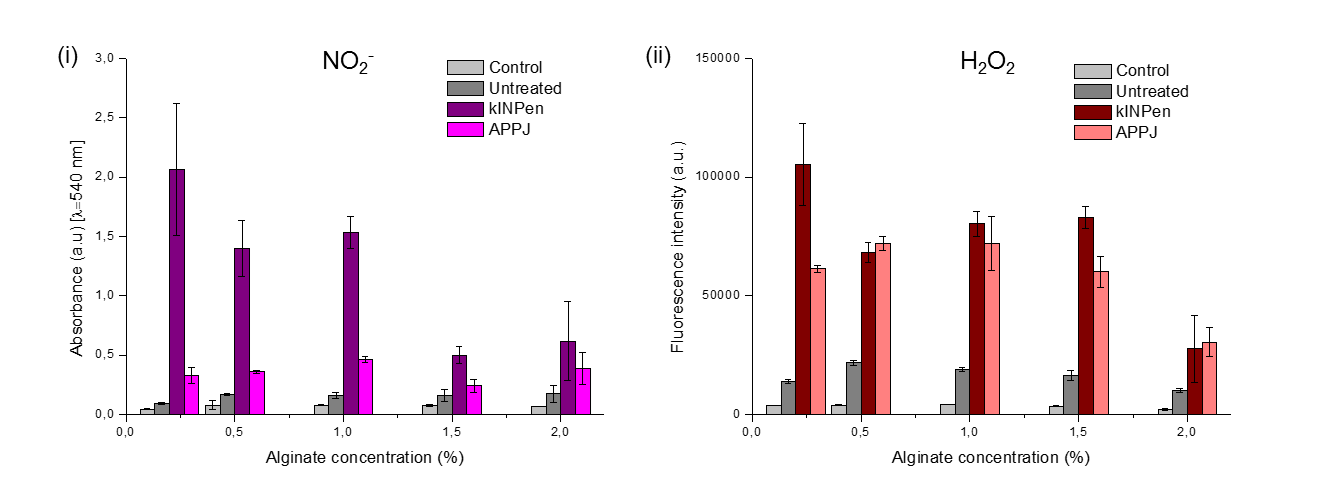


**Supplementary Figure 1**. Influence of kINPen and APPJ plasma treatment (90 s, 10 mm, 1 L/min) on different concentrations of alginate solutions: detection of nitrites (i) and hydrogen peroxides (ii). Control in grey refers to an untreated sample.





**Supplementary Figure 2**. Influence of treatment time with APPJ or kINPen on the concentration of NO_2_^-^ and H_2_O_2_ in PBS (pH 6.5) at 1 L/min gas flow and 10 mm nozzle distance.
